# Incidence and risk factors of biliary leaks after partial hepatectomy within an enhanced recovery perioperative pathway: a single-center retrospective cohort study

**DOI:** 10.1007/s00423-025-03677-w

**Published:** 2025-03-25

**Authors:** Jamy Vienet, Ismail Labgaa, Rafael Duran, Sébastien Godat, Catherine Blanc, Emilie Uldry, Emmanuel Melloul, David Fuks, Gaëtan-Romain Joliat

**Affiliations:** 1 Department of Visceral Surgery, Lausanne University Hospital CHUV, University of Lausanne (UNIL), Rue du Bugnon 46, Lausanne, 1011 Switzerland; 2 Department of Diagnostic and Interventional Radiology, Lausanne University Hospital CHUV, Lausanne, Switzerland; 3 Department of Gastroenterology and Hepatology, Lausanne University Hospital CHUV, Lausanne, Switzerland; 4 Department of Anesthesiology, Lausanne University Hospital CHUV, Lausanne, Switzerland

**Keywords:** Complications, Morbidity, Liver, Surgery, Resection

## Abstract

**Purpose:**

Biliary leak is a specific and frequent complication after hepatectomy. This study aimed to assess the incidence and risk factors of biliary leak after hepatectomy.

**Methods:**

A retrospective cohort study was performed. All consecutive patients who underwent hepatectomy between January 2013 and June 2022 were included. Abdominal drainage was performed in case of biliary anastomosis or major hepatectomy. Biliary leak was defined and classified according to the International Study Group for Liver Surgery definition with grades A, B, C based on the required management. Logistic binary regression was used to find risk factors.

**Results:**

Data were collected from 565 patients who underwent hepatectomy during the study period. Biliary leaks occurred in 10% (55/565) of patients. The rates of biliary leak grades A, B, and C were 18% (10/55), 37% (20/55), and 45% (25/55), respectively. A high nutrition risk screening (OR 2.1, 95% CI 1.3–3.4), preoperative biliary drainage (OR 4.6, 95% CI 1.5–13.5), and intraoperative biliary anastomosis (OR 3.4, 95% CI 1.3–8.9) were found as independent risk factors for biliary leak on multivariable analysis. In terms of morbidity, biliary leak patients had more infectious complications (46% vs. 8%, *p* < 0.001) and a longer median hospital stay (26 vs. 7 days, *p* < 0.001). Regarding treatment, 41 (75%) patients with biliary leak underwent drainage either endoscopically or percutaneously.

**Conclusion:**

Preoperative biliary drainage, high nutrition risk screening, and intraoperative biliary anastomosis were independent predictive factors for postoperative biliary leaks. Most frequent treatments of biliary leaks after hepatectomy were antibiotics and drainage.

## Introduction

Hepatic resection remains the current treatment of choice for a large panel of benign and malignant liver pathologies. Even though important advances in surgery, anesthesiology and perioperative management were made these past years, postoperative complications still occur in around 30–55% of cases after major hepatectomies [1–4]. One specific complication after partial hepatectomy is biliary leak [5], with variable incidence according to the type of liver resection (4–33%) [6–7]. Furthermore, it has been shown that over the years the overall morbidity rate associated with liver surgery has decreased, except for biliary leaks [8].

Biliary leak causes increased morbidity because it may require percutaneous, endoscopic, or surgical procedures [9]. Current management of bile leaks mainly involves drainage and treatment with broad-spectrum antibiotics [10], but management algorithms are scarce and heterogeneous in the current literature.

It would therefore be of importance to highlight risk factors predicting biliary leak to be able to correct them preoperatively, to adapt perioperative management, or to detect this complication earlier. Data on biliary leaks and their potential risk factors in the current era of enhanced recovery after surgery (ERAS) and minimally invasive surgery remain scant in the literature. In this context, the present study brings new detailed data on biliary leaks in patients following a standardized and homogeneous ERAS program for liver surgery. Moreover, the relationship between compliance to the ERAS program and biliary leaks is presented, which permits a more precise analysis of ERAS data.

The aim of the present study was to assess the incidence and risk factors for developing biliary leaks after partial hepatectomy in a cohort of ERAS patients.

## Methods

### Patients and eligibility criteria

The inclusion criteria were patients > 18 years old who underwent partial hepatectomy for any etiology between January 1st 2013 and June 30th 2022 in the department of visceral surgery of the Lausanne University Hospital (CHUV).

Anatomical and non-anatomical resections were performed. For primary liver cancer, anatomical partial hepatectomy was routinely performed, while for liver metastases a parenchyma-sparing approach was preferred (non-anatomical resections). Major hepatectomy was defined as resection of 4 or more hepatic segments. In case of hilar cholangiocarcinoma Bismuth II and IIIa, a right hepatectomy with resection of segment IV (extended right hepatectomy) was routinely performed and a left hepatectomy for cholangiocarcinoma Bismuth IIIb.

All consecutive patients were retrospectively collected for this cohort study. Open and minimally invasive resections were included. All patients followed an ERAS perioperative pathway based on the ERAS guidelines [11]. There were no exclusion criteria for the ERAS protocol but the compliance to the different ERAS items was measured. Compliance to the ERAS protocol was defined as the number of fulfilled ERAS items divided by the total number of ERAS items in the protocol. ERAS for liver surgery was implemented in 2013 in our department. To have a control group and the incidence of biliary leaks before ERAS (pre-ERAS cohort), data of the 100 patients who underwent liver surgery prior to ERAS implementation (2010–2012) were also collected. Preoperative biliary drainage was performed in case of total bilirubin level > 50 umol/l or of obstructed cholangitis. Abdominal drainage was performed in case of biliary anastomosis or major hepatectomy. At the end of resection, a transcystic methylene blue test was done at the surgeon’s discretion.

### Complications, biliary leaks, and definitions

The Clavien-Dindo classification was used for grading postoperative complications during the first 90 postoperative days [12]. Minor complications were defined as grades I-II and major complications as III-IV. The mortality rate (grade V) was defined by death of the patient during hospitalization or during the first 90 postoperative days. The comprehensive complication index was also calculated for all patients at 90 days after hepatectomy [13]. This index quantifies all complications of a patient (0 = no complication to 100 = death). Biliary leak was defined based on the International Study Group of Liver Surgery (ISGLS): drain bilirubin level > 3 times serum concentration at 3 days after operation or later or the necessity of a radiological, endoscopic, or surgical intervention [14]. Bile leaks were classified as grade A, B, or C based on the ISGLS definition. A biloma was defined as an extra-biliary collection of bile seen on CT-scan, magnetic resonance imaging, or ultrasound. Postoperative hemorrhage was defined according the ISGLS definition [15].

Length of stay (LoS) was calculated from operation day until day of hospital discharge.

When a biliary anastomosis was performed, a transanastomotic drain was not routinely put in place during the operation.

Nutritional status was preoperatively assessed using the nutritional risk screening (Kondrup score) [16].

### Statistical analyses

Categorical values were presented by number and percentage, while continuous values were presented using median and interquartile range (IQR). The different groups were compared using the chi-square test or the Mann-Whitney U test depending on the variable type.

To evaluate independent risk factors for biliary leak after hepatectomy, a multivariable analysis was conducted using a logistical binary regression. Age, sex, ASA score, smoking, alcohol consumption, liver disease, diabetes, portal vein embolization, preoperative biliary drainage, body-mass index (BMI), nutritional risk screening, major hepatectomy, biliary anastomosis, minimally invasive surgery, operative duration, and intraoperative blood loss were tested in the analysis. Only the items with a p-value < 0.1 on univariable analysis were included in the multivariable analysis.

Statistical analyses were performed using SPSS 29.0 (IBM Corp., Armonk, NY, USA). A p-value < 0.05 was considered statistically significant in all tests.

## Results

A total of 565 patients underwent hepatectomy during the study period. The median age of patients was 64 years old (IQR 54–71). 58% of the patients were male (328/565) and 42% were female (237/565). The median BMI was 25.1 kg/m^2^ (IQR 22.4–28.1). Major hepatectomy was performed in 202 patients (36%), and 55 patients had a biliary anastomosis (10%). Minimally invasive techniques were performed in 156 patients (28%). A total of 300 patients developed at least one complication (morbidity rate 300/565 = 53%) during the first 90 postoperative days. Biliary leaks occurred in 55 patients (55/565 = 10%). Mortality (Clavien grade V) occurred in 1% (5/565) in the entire cohort. Median LoS was 8 days (IQR 6–12).

### Preoperative characteristics, intraoperative data, and postoperative outcomes

Demographics and preoperative characteristics of patients with and without biliary leaks are summarized in Table 1. Patients with and without biliary leaks presented similar characteristics except for the numbers of active smokers, preoperative biliary drainages, and nutritional risk screenings that were higher in the biliary leak group.

**Table 1:** Preoperative characteristics of patients with and without biliary leaks

	Biliary leaks (*n* = 55)	No biliary leaks (*n* = 510)	*p*-value
Age, years°	62 (56–71)	64 (53–71)	0.671
Sex (women)	21 (38)	216 (42)	0.551
Body-mass index, kg/m^2^°	25.0 (23–28)	25.1 (23–28)	0.901
Active smoker	23 (42)	131 (26)	**0.005**
Alcohol consumption	9 (16)	87 (17)	0.896
Diabetes mellitus	11 (20)	80 (16)	0.408
Preoperative biliary drainage	10 (18)	10 (2)	**< 0.001**
Portal vein embolization	13 (24)	75 (15)	0.082
ECOG score > 2	9 (16)	7 (1)	**< 0.001**
NRS score 0–1	36 (65)	433 (85)	**< 0.001**
ASA score 1–2	34 (62)	358 (70)	0.200
Chronic liver disease Cirrhosis Viral hepatitis Alcoholic liver disease Metabolic liver disease Other liver disease	34 (62) 1 (2) 0 1 (2) 1 (2) 31 (56)	259 (51) 30 (6) 13 (3) 3 (1) 20 (4) 193 (38)	0.120 0.208 0.231 0.301 0.433 **0.008**
Hepatocellular carcinoma	4 (7)	66 (11)	0.225
Intrahepatic cholangiocarcinoma	8 (14)	44 (9)	0.149
Hilar cholangiocarcinoma	12 (21)	10 (2)	**< 0.001**
Colorectal metastases	9 (16)	220 (43)	**< 0.001**

ECOG: Eastern cooperative oncology group, NRS: nutritional risk screening, ASA: American Society of Anesthesiologists

Data are presented using median (interquartile range)° or number (percentage)

Table 2 summarizes the intra- and postoperative outcomes of patients with and without biliary leaks. In the group with biliary leak, operation time was longer (*p* < 0.001), blood loss higher (*p* < 0.001), and open surgery (*p* < 0.001), biliary anastomoses (*p* < 0.001) and abdominal drainages (*p* < 0.001) more frequent. As for the postoperative characteristics, higher rates of reoperation (*p* < 0.001), infectious complications (*p* < 0.001), surgical site infections (*p* < 0.001) and longer hospital stay (*p* < 0.001) were found in the biliary leak group.

**Table 2:** Intraoperative and postoperative outcomes of patients with and without biliary leaks

	Biliary leaks (*n* = 55)	No biliary leaks (*n* = 510)	*p*-value
Operation time, min°	361 (276–418)	250 (172–335)	**< 0.001**
Blood loss, ml°	1000 (600–1200)	500 (200–1000)	**< 0.001**
Blood transfusion	24 (44)	143 (28)	**0.016**
Biliary anastomoses	19 (39)	36 (7)	**< 0.001**
Major hepatectomy	34 (62)	168 (33)	**< 0.001**
Concomitant non hepatic procedure	3 (8)	52 (10)	0.725
Pedicular clamping	26 (47)	29 (9)	0.345
Duration of pedicular clamping, min°	21 (15–49)	25 (15–39)	0.624
Mini-invasive approach	3 (8)	153 (30)	**< 0.001**
Abdominal drainage	40 (73)	203 (40)	**< 0.001**
Major complication (IIIa-V)	48 (87)	91 (18)	**< 0.001**
90-days CCI°	42.7 (34–64)	0 (0–23)	**< 0.001**
Hemorrhage*	4 (7)	19 (4)	0.209
Infectious complication	25 (46)	39 (8)	**< 0.001**
Surgical site infection	11 (20)	15 (3)	**< 0.001**
Length of stay, days°	26 (17–37)	7 (5–11)	**< 0.001**

CCI: Comprehensive Complication Index

Data are presented using median (interquartile range)° or number (percentage)

* Hemorrhage was treated with radiological intervention or reoperation in 15 patients (3%) and transfusions only in 8 patients (1%)

Patients who had preoperative biliary drainage (*n* = 20) had similar post-drainage total bilirubin levels in the biliary leak and non biliary leak groups (14 umol/l, IQR 6–42 vs. 15 umol/l, IQR 9–26, *p* = 0.327). Thirteen patients had a repeated radiological evaluation after drainage and before the operation. Eleven patients had improvement of the biliary dilation (5 in the biliary leak group and 6 in the non biliary leak group), while 2 patients kept a dilation of the bile ducts (both in the biliary leak group, *p* = 0.515).

Pre- and intraoperative independent risk factors for biliary leaks were preoperative biliary drainage (OR 4.6, 95% CI 1.5–13.5), high nutritional risk screening (OR 2.1, 95% CI 1.3–3.4), and intraoperative biliary anastomosis (OR 3.4, 95% CI 1.3–8.9) on multivariable analysis (Table 3).

**Table 3:** Multivariable binary logistic analysis of preoperative independent risk factors for biliary leak after hepatectomy

	Univariable OR (95% CI)	*P*-value	Multivariable OR (95% CI)	*P*-value
Age, years	1.0 (1.0–1.0)	0.557		
Sex (ref: woman)	0.8 (0.5–1.5)	0.552		
ASA score	1.4 (0.8–2.4)	0.214		
Smoker (ref: no)	1.3 (0.9–1.8)	0.128		
Alcohol consumption (ref: no)	1.0 (0.6–1.6)	1		
Liver disease (ref: no)	1.6 (0.9–2.8)	0.129		
Diabetes (ref: no)	1.3 (0.7–2.7)	0.410		
PVE (ref: no)	1.6 (0.8–3.3)	0.156		
Biliary stenting (ref: no)	11.1 (4.4–28.1)	**< 0.001**	4.6 (1.5–13.5)	**0.005**
BMI, kg/m^2^	1.0 (1.0–1.0)	0.972		
Kondrup score (NRS)	2.5 (1.6–3.9)	**< 0.001**	2.1 (1.3–3.4)	**0.004**
Major hepatectomy (ref: minor)	2.7 (1.5–4.8)	**< 0.001**	1.3 (0.6–2.8)	0.449
Biliary anastomosis (ref: no)	7.5 (3.8–14.7)	**< 0.001**	3.4 (1.3–8.9)	**0.011**
Mini-invasive approach (ref: open)	0.3 (0.1–0.7)	**0.006**	0.6 (0.2–1.5)	0.243
Hilar cholangiocarcinoma (ref: no)	11.3 (4.7–27.6)	**< 0.001**	2.0 (0.5–7.6)	0.316
Operative time, minutes	1.0 (1.0–1.0)	**< 0.001**	1.0 (1.0–1.0)	0.978
Blood loss, ml	1.0 (1.0–1.0)	**0.009**	1.0 (1.0–1.0)	0.342

ASA: American Society of Anesthesiologists, PVE: portal vein embolization, BMI: body-mass index, NRS: nutritional risk screening, OR: odds ratio

### Biliary leaks

Biliary leaks occurred in 22/363 patients (6%) after minor hepatectomy and in 33/202 patients (16%) after major hepatectomy (*p* < 0.001). Among the 55 biliary leaks, 19 occurred from the biliary anastomosis site and 36 were peripheral biliary leaks (from the hepatic surface cut). Out of 55 patients with biliary leaks, 13 patients had a methylene blue test at the end of resection (6 of them had a positive test and a suture was performed). Etiologies of patients with biliary leaks (*n* = 55) were hilar cholangiocarcinoma (*n* = 12, all had a biliary anastomosis), metastases (*n* = 12), intrahepatic cholangiocarcinoma (*n* = 8, 2 had a biliary anastomosis), hepatocellular carcinoma (*n* = 4, 1 had a biliary anastomosis), and benign pathologies (*n* = 19, 4 had a biliary anastomosis). Among the 19 benign patients with biliary leaks, 9 patients were operated on for alveolar echinococcosis (3 major hepatectomies). The rates of biliary leak grade A, B, and C were 18% (10/55), 37% (20/55), and 45% (25/55), respectively. Fifty patients with biliary leaks (91%) received antibiotics for this specific complication. Drainage was performed in 41 patients: 18 percutaneously, 8 endoscopically, and 15 both percutaneously and endoscopically. In case of biliary leak, drains were left in place for a median duration of 14 days (IQR 10.5–23.5). In 36 cases, biliary leaks lead to bilomas that were found on postoperative imaging (3 were treated only with antibiotics).

Twenty-five patients had to be reoperated on due to biliary leaks (4%): 21 were operated after biliary drainage for persisting leak and 4 were operated upfront. Bacteria were found in the bile cultures in 62%, where multiple species were found most of the time. The most frequent bacteria found were *Enterococcus faecium* (*n* = 15) followed by *Enterococcus cloacae* (*n* = 6), *Escherichia coli* (*n* = 5), and *Citrobacter freundii* (*n* = 5). Hemocultures were positive in 9 patients (4 patients had both positive bile and blood cultures). In the 4 patients with both positive bile and blood cultures, the bacteria found were *Enterococcus cloacae* (*n* = 2), *Klebsiella pneumoniae*, and *Enterococcus faecium*.

Patients with high ERAS compliance (> 70%, *n* = 201) developed biliary leaks less frequently than patients with low ERAS compliance (*n* = 355, 12/201 vs. 43/355, *p* = 0.020).

In the subgroup of peripheral biliary leaks (*n* = 36), most patients were men (24/36). Three patients had preoperative biliary stenting. Major hepatectomy was performed in 17 patients. The most common etiology was colorectal liver metastases (*n* = 9). Eight patients had to be reoperated on due to these peripheral biliary leaks.

### Subgroup analyses

In the subgroup of patients with anatomical resections (*n* = 440), biliary leaks occurred in 54 patients (11 type A, 19 type B, 24 type C). Seventeen patients had preoperative biliary drainage (*n* = 10 who developed biliary leak).

Incidence of biliary leaks in the major hepatectomy cohort (*n* = 202) was 17% (34/202). Type A, B, C biliary leaks occurred in 6, 11, and 17 patients, respectively. Preoperative biliary drainage was performed in 13 patients in this subgroup. Among these 13 patients, 7 had a biliary leak.

Regarding patients with open resections (*n* = 406), 52 patients developed biliary leaks (type A: *n* = 10, type B: *n* = 19 and type C: *n* = 23). Twenty patients had a preoperative biliary drainage, while half of them (*n* = 10) had a biliary leak.

Among patients with hepatectomy without biliary procedures (*n* = 510), biliary leaks occurred in 36 patients. Six patients had type A biliary leaks, 15 type B biliary leaks, and 15 type C biliary leaks. In these 510 patients, 8 patients had a preoperative biliary drainage and 3 among them developed biliary leak.

### Pre-ERAS group

Among the 100 patients who underwent partial hepatectomy prior to ERAS implementation (median age: 64, IQR 57–70, women: 40%, median BMI: 25 kg/m^2^, IQR 22–28, major hepatectomy: 45%), overall postoperative morbidity was 65% (vs. ERAS group: 300/565 = 53%, *p* = 0.041). The rates of biliary leaks in the pre-ERAS and ERAS cohorts were similar (11/100 vs. 55/565, *p* = 0.697).

## Discussion

In the present cohort, biliary leaks occurred in 10% of patients after partial hepatectomy. Preoperative malnutrition, preoperative biliary drainage, and intraoperative biliary anastomosis were found as independent risk factors for postoperative biliary leak.

The rates of the different biliary leak grades largely vary in the literature (grade A: 2.2-9.5%, grade B 3.4–27.4%, grade C 0-4.1%) [6–7, 17]. In the present study, slightly more grade C biliary leaks were found than in the recent literature. One of the reasons already advanced by Spetzler et al. [18] is because the threshold for an intervention or an operation is specific to a center. If management is primarily based on radiological drainage or if a more aggressive management with surgery is routinely performed, the gradings will be influenced. In the present cohort, a majority of the operated cases (16/25) were reoperated during the first 5 years of the study period. Moreover, 21 of the reoperated patients first had a percutaneous drainage but needed an operation because of persisting leak. Regarding the median drainage time (14 days), it appears to be lower than in centers where a more conservative approach is performed [18]. In the end, the difference mainly depends on the management algorithm of biliary leaks.

Biliary drainage and a high nutritional risk score were both independent preoperative risk factors to develop a biliary leak after hepatectomy. This is concordant with what is found in the recent literature [19]. Malnutrition is a well-known risk factor to develop postoperative complication after hepatectomy. For biliary leak, the hypothesis is that malnourished patients do not have the required nutritional and caloric resources to maintain biliostasis [20]. Regarding preoperative biliary drainage in case of intrahepatic or hilar cholangiocarcinoma, the pathophysiological mechanism is more controversial. Some studies have shown that preoperative biliary stenting is a risk factor for biliary leak only if hyperbilirubinemia persists after drainage [21], while other studies found that biliary stenting was per se an independent risk factor [22].

In the current literature, the most common intraoperative characteristics associated with biliary leaks are concomitant non hepatic procedure, biliary anastomosis, pedicular hepatic clamping, operation time, blood loss, and blood transfusion [8, 10]. Only biliary anastomosis was found to be an intraoperative predictor of biliary leak in the present study. Zimmiti et al. showed that biliary anastomosis is a surrogate marker of a more complicated liver resection, and therefore the risk of complications and of biliary leak is higher [23, 24].

As for the postoperative part, patients with biliary leaks had a higher number of major complications, more infectious complications, and longer LoS. Similar outcomes were found in different studies, except that no significant difference was found in this study in the rate of hemorrhage between the two groups [24].

Nineteen patients developed biliary leak after biliary anastomoses (out of 55 biliary anastomoses). This rate might seem relatively high, but it should be considered that patients who underwent biliary anastomoses were a cohort potentially more at risk of complication (42% with BMI > 25 kg/m^2^, 15% with diabetes, 33% with preoperative biliary drainage, 25% with NRS > 3, and 40% with hilar cholangiocarcinoma). Moreover, complications were prospectively collected and verified during the weekly morbidity conference of the division, which permitted to obtain an accurate reporting of the incidence of biliary leaks.

No official guidelines for the management of biliary leaks after hepatectomy exist. Most of the studies are related to biliary leaks after cholecystectomy. The usual recommended strategy is a step-up approach, first with conservative treatment and afterwards radiological or endoscopic drainage or surgery if necessary. Our results showed that almost the same numbers of patients were treated by operative and non-operative methods (25 operative vs. 30 non operative). It should be kept in mind that in case of uncontrollable biliary leaks, reoperation is harder because of dense vascular adhesions which render the identification of the leaking duct and the biliostasis difficult to achieve while percutaneous drainage often can still be achieved [25]. Biliary drainage was performed in 41 cases out of 55 patients with biliary leaks (including 10 grade A biliary leaks), meaning that almost every patient had a radiological treatment in first intention before going to surgery in a step-up approach manner as described in other studies [7, 26, 27]. In terms of antibiotic therapy, 91% of patients received antibiotics, whereas only 62% had a proven infection with positive bile culture and/or positive hemoculture. Bearing in mind that the World Society of Emergency Surgery guidelines for management of bile duct injury post cholecystectomy [28] recommend antibiotic therapy only in cases of clear cholangitis or positive bile cultures (except for severe sepsis or shock), it could suggest that the administration of antibiotics was overprescribed. Clear predictive factors or specific inflammatory marker thresholds still need to be defined to identify cholangitis or infected bilomas.

This study has several limitations that need to be mentioned. Data were collected via patient records that can contain erroneous data or missing information, and collection bias can also occur. Moreover, as data originated from only one center, this can induce a selection bias. However, this study reflects the outcomes after partial hepatectomy of a homogeneous cohort of patients following an enhanced recovery after surgery pathway. Due to the relatively low number of biliary leaks, it was not possible to do subgroup multivariable analyses based on the site of the biliary leak (hepatic surface or biliary anastomosis).

In conclusion, bile leaks remain an important concern after hepatectomy, occurring in 10% in the present cohort. Potential modifiable risk factors, such as preoperative malnutrition and biliary drainage should be further assessed in the future. Nutritional prehabilitation and a selective strategy for preoperative biliary drainage could potentially improve the incidence and morbidity of biliary leaks.

## Data Availability

The datasets generated and/or analyzed during the current study are available from the corresponding author on reasonable request.
